# Assessing the postnatal condition: the predictive value of single items of the Apgar score

**DOI:** 10.1186/s12887-025-05565-0

**Published:** 2025-03-19

**Authors:** Lars Mense, Sara Nögel, Maxi Kaufmann, Helmut Küster, Nicole Braun, Burkhard Simma, Mario Rüdiger

**Affiliations:** 1 Division of Neonatology & Pediatric Intensive Care, Department of Pediatrics, Faculty of Medicine and University Hospital Carl Gustav Carus, Technische Universität Dresden, Fetscherstrasse 74, 01307 Dresden, Germany; 2 Saxony Center for Feto/Neonatal Health, Technische Universität Dresden, Dresden, Germany; 3 Department of Pediatric Cardiology, Intensive Care and Neonatology, Universitätsmedizin Göttingen, Göttingen, Germany; 4 Klinikum Westbrandenburg, Campus Potsdam, Clinic of Pediatric and Adolescent Surgery, Potsdam, Germany; 5 Department of Pediatrics, Landeskrankenhaus Feldkirch, Feldkirch, Austria

**Keywords:** Resuscitation, Newborn assessment, Apgar score

## Abstract

**Background:**

The assessment of the newborn after birth is fundamental for identifying those requiring resuscitation. Certain components of the Apgar score are used to assess neonatal condition, but their value is insufficiently validated. We aimed to identify the components of the Apgar score that predict neonatal resuscitation.

**Methods:**

Individual patient data from two multicenter trials were analyzed. Preterm newborns under 32 weeks of gestation and term newborns with perinatal acidosis and/or resuscitation were included. The extent of resuscitation was quantified by a standardized scoring system, and the clinical condition was quantified by a specified Apgar score. Correlation, linear regression and ROC analyses were used to address the study question.

**Results:**

A total of 2093 newborns were included. Newborns in poor clinical condition at 1 min received more interventions at 5 and 10 min. Heart rate, muscle tone, reflexes and breathing quality, but not skin color, were moderately strong correlated with the extent of resuscitation at 5 (*r* = 0.44 to 0.52) and 10 min (*r* = 0.38 to 0.42). Heart rate, reflexes and chest movement at 1 min best predicted the subsequent need for resuscitation (R^2^ = 0.31). The rare interventions of intubation, chest compressions and epinephrine administration can be predicted by the newborn’s condition at one minute, with a high sensitivity of 84% (cutoff ≤ 4 Apgar points) or a high specificity of 86% (cutoff ≤ 7 Apgar points).

**Conclusions:**

The clinical impression at 1 min can help to predict the need for medical interventions. Contrary to recent guidelines, heart rate, reflexes and chest movement seem to have the highest values.

**Trial registration:**

The Test APGAR study was registered at clinicaltrials.gov (NCT00623038, 14/08/2008).

**Supplementary Information:**

The online version contains supplementary material available at 10.1186/s12887-025-05565-0.

## Background

Assessing a newborn’s condition immediately after birth is fundamental for identifying neonates requiring support during postnatal transition. Appropriate postnatal support requires skilled caregivers; thus, early identification of newborns likely to require resuscitation is crucial to provide appropriate expertise in a timely manner.

In 1953, Virginia Apgar introduced a structured postnatal assessment in order to pay attention to the baby and to detect newborns in need for support, which was later named the Apgar score [1] and is assigned to newborns in almost every country around the world nowadays. In contrast to its worldwide application for half a century, recent guidelines do not recommend the Apgar score but recommend only certain components to assess neonatal conditions: heart rate, breathing and tone [2]. However, neither these three components nor the entire Apgar score have been validated for their ability to sufficiently identify newborns requiring resuscitative interventions, as per recent guidelines.

Since the original Apgar score has relevant interrater variability [3, 4] and its components are affected by resuscitation [5], a specification of the five parameters was introduced a decade ago with the aim to overcome these limitations [6]. To adapt the assessment to the current management in the delivery room, the definitions of the five items of the Apgar score were specified to document the neonatal condition independently of the measures taken to achieve this condition and with respect to gestational age [6].

Furthermore, the American Academy of Pediatrics (AAP) and the American College of Obstetricians and Gynecologists (ACOG) recommended reporting interventions for neonatal resuscitation in a standardized form [7]. This recommendation has been used to quantify resuscitative interventions in the delivery room [8–11]. Both the assessment of the infant´s condition with the specified items of the Apgar score and the quantification of resuscitative interventions according to AAP and ACOG recommendations have been shown to predict the outcomes of newborns in two prospective clinical trials [9–11].

## Methods

### Aim of the study

Using the specified assessment of the infant`s condition and the quantification of resuscitative interventions, we aimed to address the following objectives: Primarily, does the infant’s condition 1 min after birth predict the need for neonatal resuscitation at 5–10 min? Secondarily, which item predicts the extent of resuscitation best? Can rare events of neonatal resuscitation, such as intubation, chest compressions and the administration of epinephrine, be predicted at 1 min of life, so that more experienced caregivers can be called immediately?

### Study design

We performed a post hoc, secondary analysis of two prospective, observational trials. The trials were performed in two neonatal populations that are most likely to require resuscitative interventions: The TEST-Apgar trial studied very preterm infants (< 32 weeks of gestation) and was performed at 20 academic neonatal intensive care units in 12 countries [9]. The second observational trial included term newborns requiring resuscitation or having an umbilical cord pH < 7.00 studied in six neonatal intensive care units in Germany.

### Definitions

Both studies were performed under routine clinical care conditions. Caregivers used the specified definitions to assess the infant’s condition and quantify the extent of resuscitation at one, five and ten minutes of life, which have been published in extent previously [9]. The infants’ conditions were scored regardless of the interventions needed to achieve the condition. In detail, reflexes and muscle tone were assessed according to their appropriateness for the infant’s gestational age as judged by the responsible caregiver, i.e.2 points were assigned for appropriate reflexes, 1 point for reduced and 0 points for absent reflexes. For respiration, infants received a score of 0 if no chest movement was found, 1 for inappropriate chest movement, and 2 for appropriate chest movement, regardless of their respiratory support. Heart rate and skin color were scored as described by Virginia Apgar, again regardless of the medical intervention. To quantify the extent of neonatal resuscitation, the following interventions were scored: continuous positive airway pressure (CPAP), oxygen supplementation, bag and mask ventilation, intubation and ventilation, chest compressions, surfactant and drugs (epinephrine). One point was given for each intervention applied. A detailed set of definitions can be found in Supplemental Tables 1 and 2.

### Statistics

The data were not normally distributed and are expressed as the median (25th; 75th percentile) for metrical data. Statistical significance was tested using the Kruskal‒Wallis test. Correlations were tested using Spearman-Rho statistics, and confidence intervals were calculated as suggested by Fieller, Hartley and Pearson [12]. Differences in correlation coefficients were assessed using Fisher Z transformations according to Sheshkin [13]. The influence of infants’ condition at 1 min on the number of interventions the infant received at 5 min was tested using stepwise linear regression analysis (alpha-to-enter of 0.05, alpha-to-leave of 0.10). Whether the infant’s condition at 1 min identifies neonates requiring interventions of neonatal resuscitation was tested using receiver operating characteristic (ROC) curve analyses. Cutoff values were suggested based on either a high sensitivity (sensitivity > 0.8) or a high specificity (specificity > 0.8). *P* < 0.05 was considered to indicate statistical significance, and the alpha level was adjusted for multiple testing according to Dunn (Kruskal‒Wallis tests) or Bonferroni (Sheshkin tests). The analyses were performed using IBM SPSS 28.0 and GraphPad Prism 9. Deidentified patient data are available at the online repository OpARA (https://opara.zih.tu-dresden.de).

## Results

### Patients

Data from 1926 newborns born at or below 32 + 0 weeks (preterm cohort, 92.0%) and 167 newborns with a gestational age of 37 + 0 weeks or above (term cohort, 8.0%) were included in the present study. The median gestational age was 29^0/7^ (27^0/7^; 31^0/7^) weeks in the preterm cohort and 39^0/7^ (37^5/7^; 40^3/7^) weeks in the term cohort, with median birth weights of 1125 (850; 1450) g and 3340 (2940; 3750) g, respectively.

### Clinical condition at one minute of life predicts the extent of resuscitative interventions

**Fig. 1:**
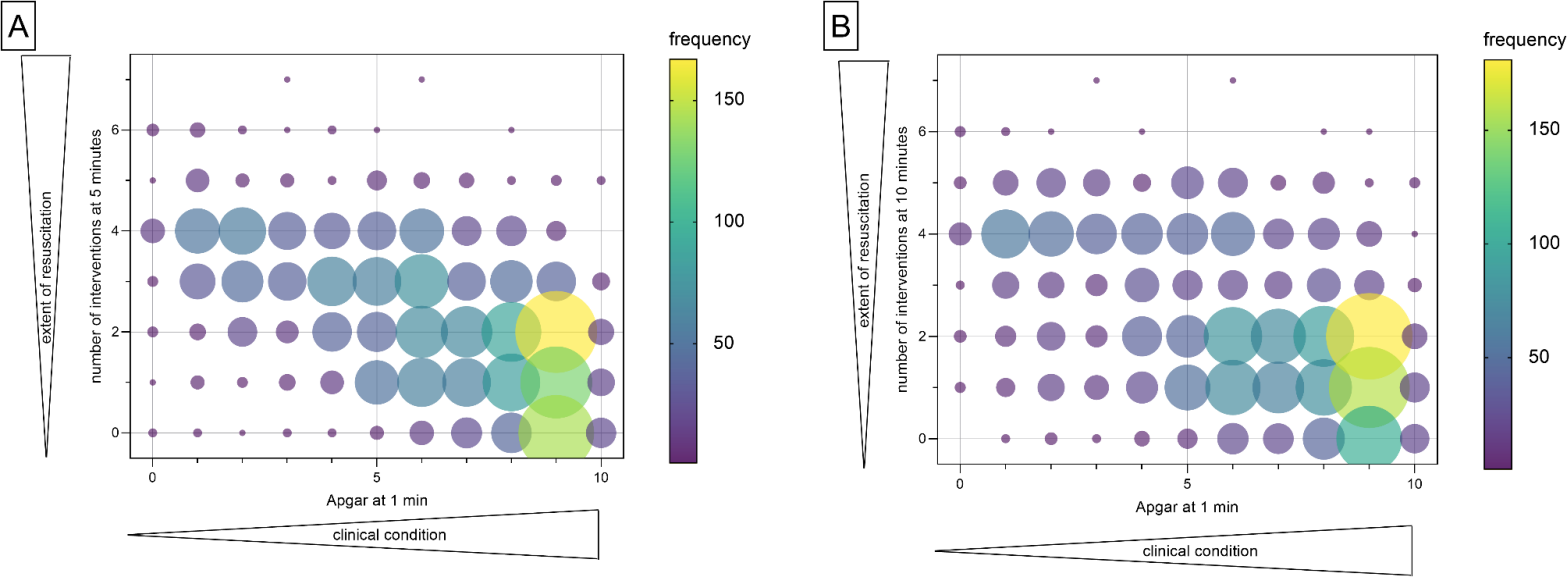
Association of the clinical condition at 1 min and the extent of neonatal resuscitation at 5 (**A**) and 10 (**B**) minutes (measured as the number of interventions). The colors and sizes of the bubbles indicate the frequency

Newborns with a poor clinical condition at one minute, represented by a lower specified Apgar score, received more interventions at 5 and 10 min of life (Fig. 1A and B). Similarly, newborns with a lower score at 5 min received more intense resuscitation at 10 min (Supplemental Fig. 1).

**Fig. 2:**
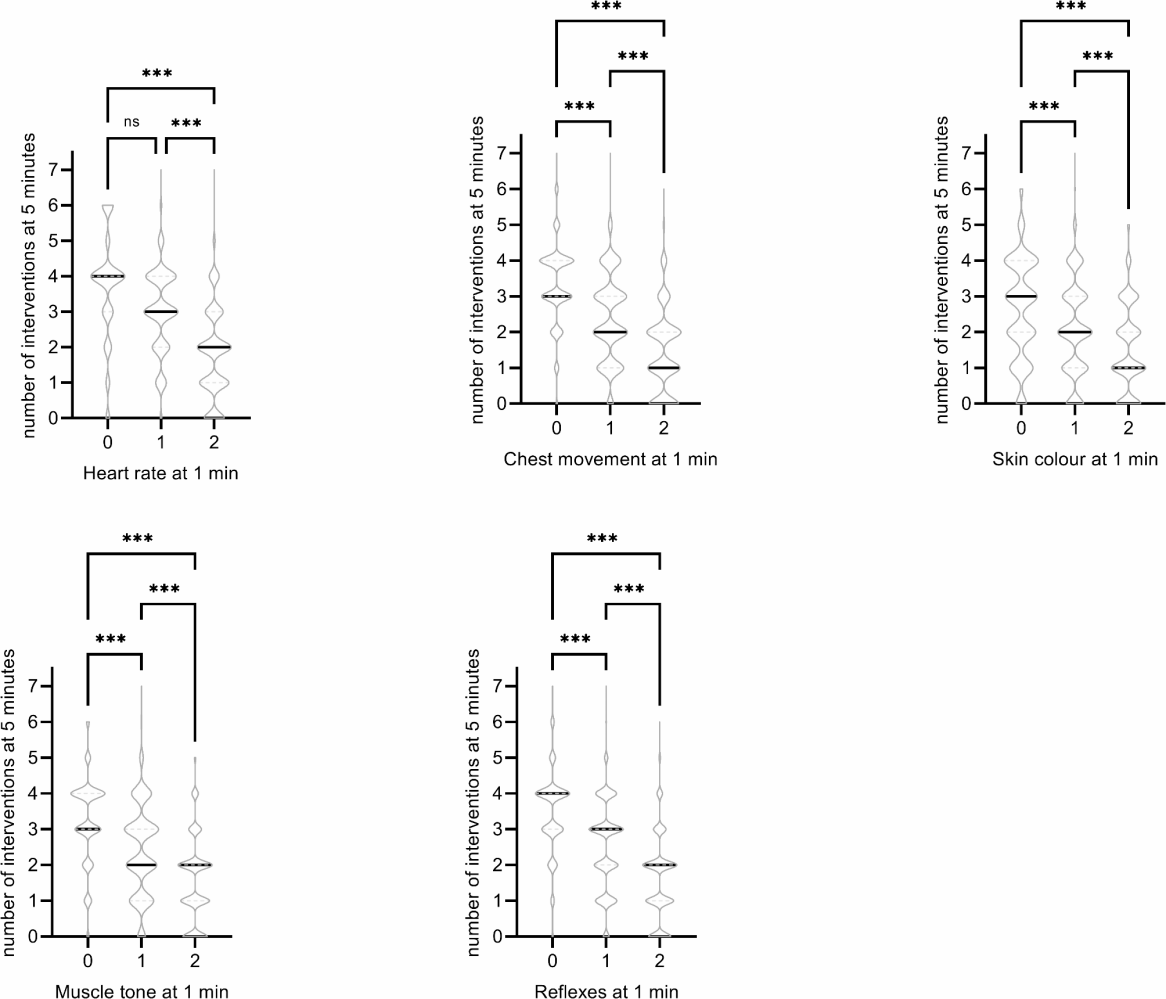
Number of resuscitative interventions at 5 min in relation to single items of infant’s condition at 1 min. * *p* < 0.05, ** *p* < 0.01, *** *p* < 0.001

A detailed analysis revealed that lower scores on any of the five specified Apgar items at 1 min were associated with more resuscitation at 5 min of life (Fig. 2). Four of the five specified items of the Apgar score demonstrated moderately strong correlations with the extent of neonatal resuscitation at 5 min and, at lower strength, with the extent of resuscitation at 10 min of life (Table 1, Supplemental Table 3).

**Table 1:** Correlation between the infant’s condition and the number of interventions

		Interventions	
		At 5 min	At 10 min
Condition at 1 min	Heart rate	0.444 (0.408–0.479) #	0.384 (0.346–0.422)
	Skin colour	0.213 (0.170–0.255) *	0.212 (0.169–0.255) *
	Chest movement	0.515 (0.481–0.546)	0.423 (0.386–0.459)
	Reflexes	0.492 (0.458–0.525)	0.414 (0.377–0.451)
	Muscle tone	0.459 (0.423–0.493)	0.374 (0.355–0.412)

Correlation coefficients with 95%-confidence intervals between the infant’s condition at 1 min and the number of interventions at 5 and 10 min of life. All correlations are statistically significant (*p* < 0.001). * *p* < 0.005 compared to the correlations between the interventions and all other conditions at 1 min. # *p* < 0.005 compared to the correlation between the interventions at 5 min and chest movement at 1 min. Exact p-values can be found in Supplemental table S3.

Linear regression analyses with the number of interventions at 5 min as the dependent variable and single items of the specified Apgar score at 1 min as the independent variable revealed that heart rate, reflexes and chest movement at 1 min significantly explained the variability of resuscitative interventions at 5 min: 31% of the variability is explained by the three variables (F3,2064) = 306.868, *p* < 0.001). The regression coefficients were − 0.47 (heart rate), -0.38 (reflexes) and − 0.48 (chest movement).

Similar results were found for resuscitative interventions at 10 min: 23.8% of the variability was explained by heart rate (-0.51), reflexes (-0.41) and chest movement (-0.36) at 1 min (F3,2030) = 212.890, *p* < 0.001). A similar regression model with single items of the Apgar score at 5 min was not better at predicting the need for resuscitation at 10 min (R^2 of 0.246).

### The infant’s condition predicts the need for severe neonatal resuscitation

Intubation, chest compressions and epinephrine administration are rare events of neonatal resuscitation, and skilled providers are required to provide them safely. The newborn’s condition at 1 min of life predicts the subsequent need of these interventions (Table 2).

**Table 2:** Area under the curve of ROC analyses for the prediction of rare interventions

	Infant’s condition at 1 min		Heart rate, reflexes, chest movement at 1 min	
	Intervention at 5 min	Intervention at 10 min	Intervention at 5 min	Intervention at 10 min
Chest compressions	0.83 (0.77–0.89)	0.80 (0.70–0.91)	0.82 (0.75–0.88)	0.80 (0.68–0.91)
Intubation	0.78 (0.76–0.80)	0.79 (0.76–0.81)	0.78 (0.76–0.80)	0.79 (0.77–0.81)
Epinephrine	0.89 (0.83–0.95)	0.88 (0.80–0.96)	0.88 (0.82–0.94)	0.91 (0.83–0.98)

Area under the curve (AUC) of the ROC analyses with 95%-confidence intervals for predicting rare interventions of neonatal resuscitation by the newborn’s condition at 1 min.

An Apgar score of 4 or less at 1 min identified newborns requiring one or more of these interventions at 5–10 min of life with a specificity of 86%, although the sensitivity was low (51%) (Supplemental Table 4). A cutoff of 7 or less identified these newborns with high sensitivity (84%, specificity 52%). In our cohort, the positive and negative predictive values (PPV, NPV) were 61% and 79% (cutoff of 4 or less) and 45% and 86% (cutoff of 7 or less), respectively.

When only the three items identified earlier (heart rate, reflexes, chest movement) were used, the results of the ROC analysis were similar (Table 2). For these three items, a score of two or less predicts the need for either intubation, chest compression or epinephrine administration with a sensitivity of 43% and specificity of 91% (PPV 68%, NPV 77%).

## Discussion

### Main findings

To our knowledge, the present study is one of the first to evaluate whether specified items of an infant’s condition immediately after birth predicts the need for subsequent resuscitative interventions in modern neonatology. We demonstrated that the need for and extent of support can be predicted by assessing an infant’s condition at one minute of life, with heart rate, chest movement and reflexes being the most important features. Furthermore, the infant’s condition at one minute identifies the need either for caregivers experienced in basic neonatal life support or for more experienced caregivers since rare interventions might be necessary within the next minutes.

### Interpretation

The identification of newborns in need of extensive resuscitation is important since it is a rare event among newborn infants [14, 15]. Since the level of experience among care providers for very preterm infants might vary in between centers and internationally, an early identification of newborns requiring rare interventions is therefore important.

Current resuscitation guidelines utilize three components of the infant’s condition (respiration, heart rate and tone) to identify newborns likely needing resuscitation [2]; however, scientific evidence to support these recommendations is lacking. Our analysis is the first to assess how good single items of infant’s condition predict the extent of subsequent resuscitation. To overcome the limitations of the conventional approach to describe an infant’s condition, such as high interrater variability of the Apgar score and interference with resuscitative interventions [3–5], we used a well-evaluated specification of the single items of the Apgar score [9–11]. The specified Apgar score has the advantage of describing an infant’s condition even during ongoing resuscitative interventions and regardless of gestational age. The particular definitions used for the specified Apgar score should be considered by the caregiver. Furthermore, the extent of neonatal resuscitation was quantified in accordance with the recommendations of the AAP and ACOG [7]. Since the seven most important resuscitative interventions were quantified in a standardized manner, we were able to analyze how good the need for single interventions (such as chest compressions) is predicted by the infant’s condition (regardless if interventions are performed or not at the time of assessment).

Other alternatives to assess the infant’s condition and resuscitative interventions have been proposed in recent years, such as the Neonatal Resuscitation and Adaptation Score (NRAS) [16]. However, the prognostic value of its single items has not been evaluated yet. Interestingly, initial grimace and activity have recently been investigated to predict the required level of respiratory support in very preterm infants [17]. Those with low combined grimace and activity scores after placement on the resuscitation bed received a greater amount of cardiovascular support. This is in line with our findings, which show that the combination of reflexes, heart rate and chest movement is predictable of support that is more extensive.

Our data suggest that, in the context of the specified Apgar score, heart rate, chest movement and reflexes are the most important parameters, Heart rate can be measured by a variety of methods, such as auscultation, electrocardiogram or palpation, with low interobserver variability [6]. Heart rate highly affects cardiac output in newborns and thereby influences other components of the Apgar score such as skin color and tone, underlining its importance. Chest movement is accompanied by active respiration or effective ventilation by the caregiver and can be difficult to assess, especially in very low birth weight infants [3]. Nevertheless, effective aeration of the lung is highly important during the feto-neonatal transition. However, because it is more difficult to assess reflex irritability, the interobserver variability is low [18]. Given our results, the assessment of muscle tone and skin color is of lower importance for the prediction of resuscitation, which is consistent with previous data [4, 19].

### Strengths and limitations

A great strength of our analysis is that it is based on a large sample size. Furthermore, the studies were performed in a prospective multicenter design, and strict methods to quantify infant’s clinical condition and extent of resuscitation were used. Nevertheless, our results are based on a secondary analysis of prospectively collected data. The two original studies did only include newborns with a high likelihood to receive resuscitative interventions. Patients between 32 and 37 weeks of gestational age were not included. The distribution of our population represents the patients that are resuscitated with about 90% being preterm and 10% being born term. Since the analysis uses infant’s condition regardless of gestational age, we believe that the results are also applicable to preterm infants between 32 and 37 weeks as well. Their clinical signs and physiological responses do not differ from other newborns. Information on cord management was not recorded in both trials. Immediate cord clamping was most likely performed in some infants and potentially influenced Apgar scores. Since the different components of the Apgar score and neonatal interventions are not affected by the cord clamping strategy, the impact on the study question is low. Heart rate was determined as per standard protocol of the units, most likely primarily per auscultation, which is in accordance with the guidelines [2]. Not all infants received an ECG since we recorded data reflecting routine care, which is a strength of our study. Muscle tone and reflexes were judged by the caregiver regarding their appropriateness for gestational age. This judgement is subjective but more defined as the original Apgar score although the interrater variability has not been formally tested yet [9].

Despite these limitations, our data are of great clinical relevance. We can provide an evidence-based approach to predict the need for subsequent resuscitative interventions in newborns within the first minute of life. Our results require the assessment of the newborn using the specified scoring system. Low scores in heart rate, reflexes and chest movement are the strongest predictors of advanced resuscitative interventions.

If all five items of the specified Apgar score are used at one minute, infants with a score higher than 7 are less likely to require advanced interventions of neonatal resuscitation (intubation, chest compression or epinephrine). However, infants with a score less than 5 are likely to need special neonatal expertise for more complex resuscitative interventions. To ease the newborn’s assessment, it would be sufficient to evaluate heart rate, reflexes and chest movement with comparable predictive value. In this case, a score of 2 or lower identifies infants likely needing special neonatal experience for the more complex interventions.

## Conclusions

The need for subsequent neonatal resuscitation can be predicted by the infant’s condition at 1 min using either all 5 specified items of the Apgar score or only its three components, heart rate, reflexes and chest movement. The condition at 1 min helps to identify newborns requiring caregivers that are more experienced.

## Electronic supplementary material

Below is the link to the electronic supplementary material.

Supplementary Material 1

Supplementary Material 2

## Data Availability

The datasets generated and/or analyzed during the current study are available in the online repository OpARA (https://opara.zih.tu-dresden.de).

## Abbreviations

AAP: American Academy of Pediatrics
ACOG: American College of Obstetricians and Gynecologists
CPAP: Continuous positive airway pressure
NPV: Negative predictive value
PPV: Positive predictive value
