# Supplementary figures and images for: Assessing the postnatal condition: the predictive value of single items of the Apgar score
Source: BMC Pediatr. 2025 Mar 19;25:214. doi: 10.1186/s12887-025-05565-0 (PMC11921496; doi:10.1186/s12887-025-05565-0)

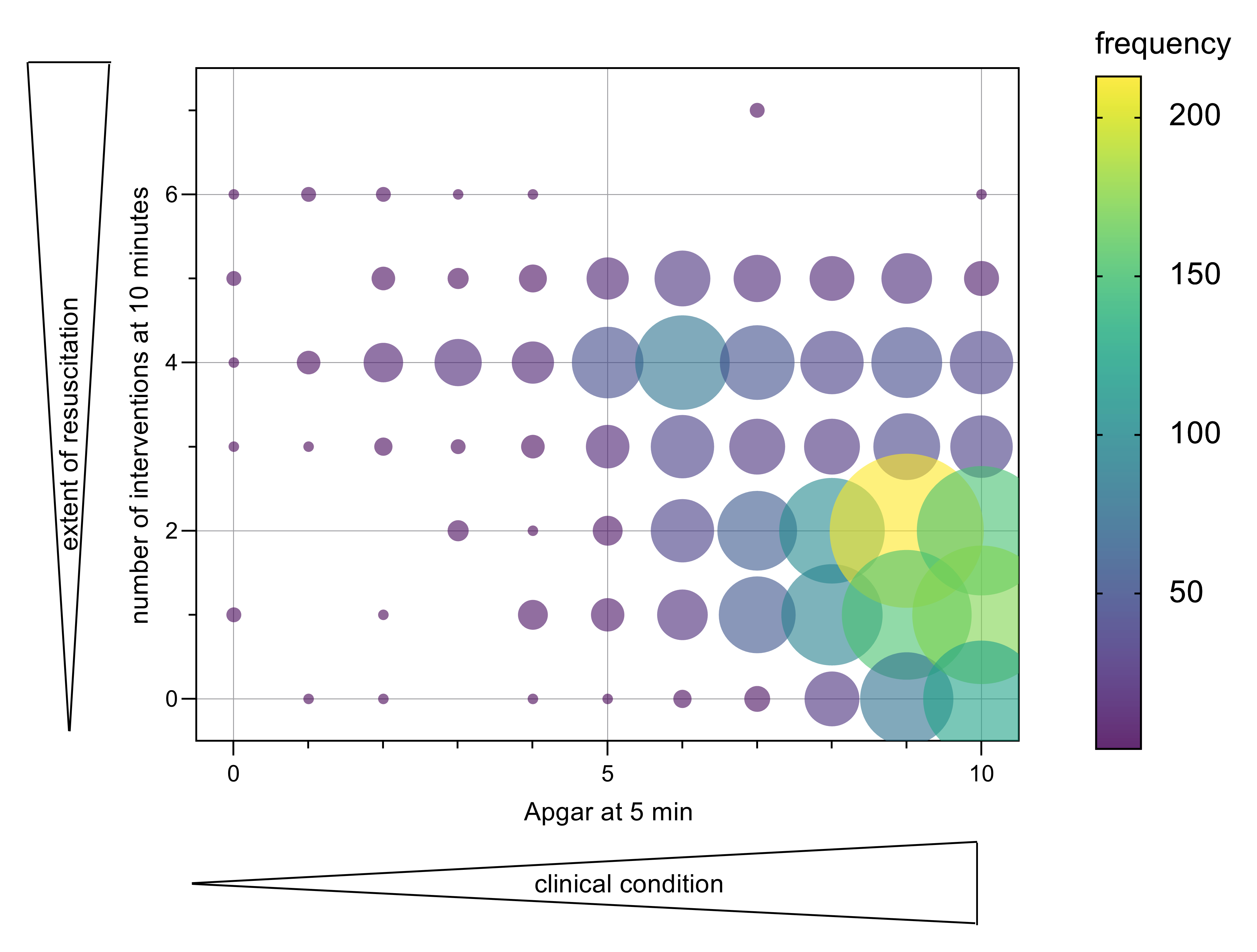

Supplement: Supplementary file 1 — Supplementary Material 1 [file 12887_2025_5565_MOESM1_ESM.tif]
