# Supplementary material for: Assessing the postnatal condition: the predictive value of single items of the Apgar score
Source: BMC Pediatr. 2025 Mar 19;25:214. doi: 10.1186/s12887-025-05565-0 (PMC11921496; doi:10.1186/s12887-025-05565-0)
Supplement: Supplementary file 2 — Supplementary Material 2 [file 12887_2025_5565_MOESM2_ESM.docx]

| A – Skin color* | 2 | Completely pink |
| --- | --- | --- |
|  | 1 | Centrally pink with acrocyanosis |
|  | 0 | Centrally blue or pale |
| P – Heart rate* | 2 | > 100 / min |
|  | 1 | 1-100 / min |
|  | 0 | No heart rate |
| G – Reflex | 2 | Appropriate for gestational age |
|  | 1 | Reduced for gestational age |
|  | 0 | No reflex responses |
| A – Muscle tone | 2 | Appropriate for gestational age |
|  | 1 | Reduced for gestational age |
|  | 0 | Completely flaccid |
| R – Chest movement* | 2 | Regular chest movement |
|  | 1 | Small or irregular chest movement |
|  | 0 | No chest movement |

Supplemental table 1. Specification of the 5 items of the Apgar score in order to meet requirements of modern neonatal resuscitation (* independent of interventions required to achieve this condition).

| CPAP | Score 1 if “Mask and bag ventilation” or “Intubation and ventilation” is scored 1. |
| --- | --- |
| Oxygen supplementation |  |
| Mask and Bag Ventilation | Mask and Bag ventilation. Score 1, if “Intubation and ventilation” is scored 1. |
| Intubation and Ventilation |  |
| Chest compressions |  |
| Exogenous surfactant administration |  |
| Drugs |  |

Supplemental table 2. Expanded Apgar score (score 1 if the intervention is performed, score

0 if the intervention is not performed).

|  | Heart rate at 1 minute | Skin colour at 1 minute | Chest movement at 1 minute | Reflexes at 1 minute |
| --- | --- | --- | --- | --- |
|  | Number of interventions at 5 minutes | | | |
| Skin colour at 1 minute | **<0.001** |  |  |  |
| Chest movement at 1 minute | **0.004** | **<0.001** |  |  |
| Reflexes at 1 minute | 0.05 | **<0.001** | 0.33 |  |
| Muscle tone at 1 minute | 0.56 | **<0.001** | 0.02 | 0.18 |
|  | Number of interventions at 10 minutes | | | |
| Skin colour at 1 minute | **<0.001** |  |  |  |
| Chest movement at 1 minute | 0.15 | **<0.001** |  |  |
| Reflexes at 1 minute | 0.27 | **<0.001** | 0.73 |  |
| Muscle tone at 1 minute | 0.72 | **<0.001** | 0.07 | 0.14 |

Supplemental table 3. p-values of the differences between Spearman rho correlation coefficients between components of the 1 minute Apgar score and the number of interventions at 5 or 10 minute. P<0.005 is deemed statistically significant (adjusted for multiple testing).

| Intervention and suggested 1 minute-specified Apgar cut-off | Sensitivity | Specificity |
| --- | --- | --- |
| Chest compression at 5 minutes  ≤ 5 (high sensitivity)  ≤ 3 (high specificity) | 0.844  0.667 | 0.655  0.827 |
| Chest compression at 10 minutes  ≤ 5 (high sensitivity)  ≤ 3 (high specificity) | 0.833  0.667 | 0.650  0.820 |
| Intubation at 5 minutes  ≤ 6 (high sensitivity)  ≤ 4 (high specificity) | 0.807  0.550 | 0.606  0.827 |
| Intubation at 10 minutes  ≤ 7 (high sensitivity)  ≤ 4 (high specificity) | 0.860  0.540 | 0.512  0.849 |
| Epinephrine at 5 minutes  ≤ 4 (high sensitivity)  ≤ 3 (high specificity) | 0.833  0.792 | 0.749  0.824 |
| Epinephrine at 10 minutes  ≤ 4 (high sensitivity)  ≤ 3 (high specificity) | 0.818  0.727 | 0.746  0.819 |
| Any rare interventions at 5 or 10 minutes  ≤ 7 (high sensitivity)  ≤ 4 (high specificity) | 0.840  0.505 | 0.524  0.857 |

Supplemental table 4. Suggested cut-off values to predict rare interventions of neonatal resuscitation by the clinical status of the newborn at 1 minute of life (measured by the specified Apgar score). Cut-off values were selected by either choosing a sensitivity of 80% or higher or a specificity of 80% or higher.
